# Repurposing natural compounds: computational evaluation of *Piper longum* alkaloids against ovarian cancer transcriptomic signatures

**DOI:** 10.3389/fbinf.2026.1872927

**Published:** 2026-06-29

**Authors:** Guruswamy Vimalkumar, Abul Kalam Azad Mandal

**Affiliations:** School of Bio Sciences and Technology, Vellore Institute of Technology, Vellore, Tamil Nadu, India

**Keywords:** EGFR, molecular docking, molecular dynamcis, network phamacology, ovarian cancer, *Piper longum*

## Abstract

**Background:**

Ovarian cancer is a deadly gynaecological cancer, diagnosed at advanced stages, resistant to chemotherapy and with low survival rates. Current treatments are ineffective, and new multi-targeted candidates are needed. The alkaloids of *Piper longum* (piperine, piperlongumine, and piperlonguminine) have reported anti-cancer effects however, specific molecular targets and underlying mechanism of action in ovarian cancer remain largely unknown.

**Methods:**

DEGs from five GEO datasets were obtained using GEO2R (|log2FC| > 1, p < 0.05) and compared with alkaloid targets from SwissTargetPrediction, GeneCards, and CTD. Protein-protein interaction PPI networks were built with STRING v12.0, and hub genes were identified with CytoHubba and MCODE. Gene ontology, GEPIA2 expression, Kaplan-Meier survival, and GSCA mutation analyses confirmed clinical significance. Binding stability was evaluated using Molecular docking (AutoDock Vina) and molecular dynamics simulations (GROMACS, 2023.1, CHARMM36m 200 ns).

**Results:**

Cross-referencing 14,063 DEGs with 483 alkaloid targets yielded 342 shared genes. PPI analysis produced a robust network (292 nodes, 6,373 edges; p = 1.0 × 10^−16^), identifying ten hub genes: TP53, CTNNB1, AKT1, IL6, TNF, EGFR, CASP3, BCL2, MYC, and JUN. Of these, BCL2, EGFR, JUN, TNF, and TP53 were significantly dysregulated in tumour tissues, with JUN and EGFR as key adverse prognostic drivers. TP53 showed the highest mutation frequency (100%). Piperine exhibited the strongest docking affinity for EGFR (−8.701 kcal/mol), and MD simulations confirmed EGFR–piperine as the most stable complex (RMSD: 0.20–0.25 nm), driven by hydrophobic anchoring.

**Conclusion:**

*P. longum* alkaloids, especially piperine, display potent multi-targeted anti-ovarian cancer activity, with EGFR as the main validated binding site. They also have better pharmacokinetic profiles than paclitaxel. Our results offer a mechanistic rationale for phytochemical repurposing in ovarian cancer and a reproducible framework for future experimental validation.

## Graphical Abstract

**Figure:**
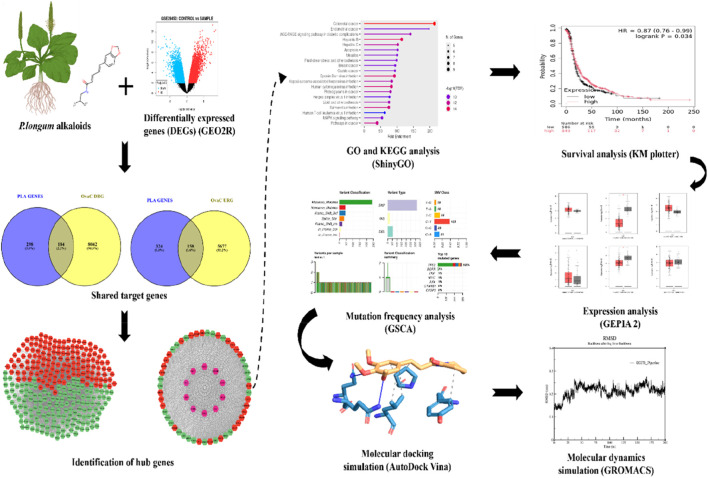

## 1 Introduction

Ovarian cancer is a life-threatening gynaecological cancer, which causes recalcitrant symptoms with high mortality in patients who are in critical condition (Fan et al., 2024). It is in the eighth position in leading cause of death in women due to cancer all around the world (Krishna et al., 2026). This is an invasive cancer that has complex molecular and genetic factors which makes it very fatal and resistant to conventional therapeutics (Aggarwal et al., 2025). The factors that contribute to ovarian cancer include environmental influences, fertility treatments, alcohol intake and genetic mutations (Wang et al., 2022).

This is a deadly and potentially fatal illness that affects women all over the world. With new cases predicted to climb by 42% by 2040, from 313,959 in 2020 to 445,721, the global increase in ovarian cancer poses a serious public health concern. In a similar vein, mortality rates are expected to increase by 51% (Muttiah and Abdullah, 2026).

It is often called as “Silent killer” due to the deceptive character of its onset, and the circumstance of its detection. This late diagnosis is linked with its enigmatic symptoms that include abdominal pain, bloating, and problems with urinating which can in many instances be mistaken with the milder health conditions (Islam et al., 2023). The common treatment that are available for this cancer include surgery, radiation and chemotherapy, these conventional treatments also have adverse side effects such as reappearance of the symptoms, metastasis and chemotherapy resistance (Wang G. et al., 2025).

The art of using plants and their components to treat several diseases among people has a history. Various medicinal plants have been seen to be helpful in improving quality of life by acting as antioxidant and anti-inflammatory as well as antidiabetic (Kanwal et al., 2022). *Piper longum,* which is commonly known as pippali or long pepper which has a very prominent role in Indian traditional medical systems like ayurveda and siddha, in which it is considered as “Rasayana herb” known for its immunomodulatory and rejuvenating properties (Bhatia et al., 2026).

The phytochemical research done on this herb revealed that amide alkaloids are the major subclass that are present, among these alkaloids present in the herb piperine is the major and biologically active alkaloid followed by piperlongumine and piperlonguminine (Henrique et al., 2020; Viet Phong et al., 2022). These alkaloids exhibit a wide range of anti-cancer activities against various cancer cell lines (Rather and Bhagat, 2018; Henrique et al., 2020; Viet Phong et al., 2022). These alkaloids modulate several oncogenic signals (P38, PI3K/Akt/mTOR, and MAPK/ERK) thereby contributing to its pro-apoptotic effects (Chang et al., 2025; Henrique et al., 2020; Singh et al., 2023). Thus, enhancing its potential as a targeted drug for cancer.

A technique known as network pharmacology, proposed by Hopkins in 2007 in Nature Biotechnology that has undergone updating as of 2011 to today’s so-called network targets, multiple-constituent method, is a new drug development methodology that involves considering the intricate biological processes and choosing particular signaling nodes to assemble multi-target drug molecules. It underlines the active constituents, enhances therapeutic properties of drugs, decreases toxicity and maps active constituents to disease gene (Kanwal et al., 2022). In this present study we determined the binding affinity of the primary alkaloids of *P. longum*, i.e., piperine, piperlongumine and piperlonguminine individually to classify the therapeutic potential of these lead alkaloids in ovarian cancer. Molecular docking was used to determine the orientation of binding specificity of each compound. To determine the stability of individual interaction, binding free energy, and conformational transitions of each alkaloid in its corresponding binding pocket of their respective proteins over time, this was to be followed by molecular dynamics simulation. This multidimensional computational modelling approach provides mechanistic insights at the enhanced resolution on the mechanism of action of these particular phytochemicals to target the nodes of signalling pathways to explain the rationale of candidate lead compounds to target ovarian cancer.

## 2 Materials and methods

### 2.1 ADME (absorption, distribution, metabolism, and excretion) and physiochemical profiling of *Piper longum* alkaloids

To assess the pharmacokinetic profile and therapeutic potential of the *P. longum* alkaloids (Piperine, Piperlongumine, and piperlonguminine), an *in silico* ADME analysis was performed. The canonical SMILES (Simplified Molecular Input Line Entry system) of the compounds were obtained from PubChem compound database (https://pubchem.ncbi.nlm.nih.gov/) and were submitted to the SwissADME server (https://www.swissadme.ch/), developed by the Swiss institute of bioinformatics. The computational tool was utilized to predict key physicochemical parameters, including molecular weight, topological polar surface area (TPSA), and the number of hydrogen bond donors and acceptors. Lipophilicity was evaluated using the consensus LogP model. Furthermore, crucial pharmacokinetic characteristics, specifically predicted gastrointestinal (GI) absorption and Blood-brain-barrier permeability, were assessed. Finally, the drug-likeness of the alkaloids was strictly evaluated based on their compliance with Lipinski’s Rule of five to confirm their potential as orally bioavailable drug candidates. Paclitaxel a well-known ovarian cancer drug was used as a control for comparison (Daina et al., 2017).

### 2.2 *In-silico* toxicity prediction

The toxicity profiles of the individual *P. longum* alkaloids (piperine, piperlongumine, and piperlonguminine) were computationally evaluated to assess their safety parameters. The ProTox-3.0 prediction server (https://tox.charite.de/protox3/) was utilized to determine the potential toxicological risks associated with these compounds. The *in silico* assessment focused on two primary categories: organ toxicity, encompassing hepatotoxicity and cardiotoxicity, and critical toxicity endpoints, which included carcinogenicity, immunotoxicity, and mutagenicity.

### 2.3 Mining of ovarian cancer transcriptomes for high-confidence DEGs

The differentially expressed genes (DEGs) in ovarian cancer patients were retrieved from the gene expression omnibus (GEO) database (https://www.ncbi.nlm.nih.gov/) (Wang G. et al., 2025) which includes GSE10971, GSE12470, GSE26712, GSE29450, GSE54388. The GSE10971 dataset comprises the gene expression data of 37 samples obtained from non-malignant fallopian tube epithelium and high-grade serous carcinoma. Although our primary focus is ovarian cancer, dataset GSE10971 which includes fallopian tube epithelium from BRCA1/2 mutation carriers was included in our analysis because these pre-neoplastic transcriptomic profiles closely resemble high-grade serous ovarian carcinoma, providing a robust model for DEG mining (Shaw et al., 2009). Among the 37 samples 24 were from normal control patients and 13 were from patients with ovarian cancer. The GSE12470 dataset comprises the gene expression data of 53 samples obtained from normal peritoneum and serous ovarian cancer. Among the 53 samples 10 were from normal control patients and 43 were from patients with ovarian cancer. The GSE26712 dataset comprises the gene expression data of 195 samples obtained from normal ovarian surface epithelium and ovarian tumor. Among the 195 samples 10 were from normal control patients and 185 were from patients with ovarian cancer. The GSE29450 dataset comprises the gene expression data of 20 samples which are taken from both normal ovarian surface epithelium and ovarian tumor. Among the 20 samples 10 are taken from normal control patients and 10 are from patients with ovarian cancer. The GSE54388 dataset comprises the gene expression data of 22 samples obtained from normal ovarian surface epithelium and ovarian tumor epithelium. Among the 22 samples 6 were from normal control patients and 16 were from patients with ovarian cancer.

The DEGs between ovarian cancer patients (sample) and healthy individuals (control) were identified using the GEO2R interactive web tool (https://www.ncbi.nlm.nih.gov/geo/geo2r/) with selection criteria of P < 0.05 and |logFC| > 1. The P- values were adjusted using the Benjamini–Hochberg false discovery rate (FDR) (Wang G et al., 2025). The functional annotation of the DEGs were done by a web based interface g:profiler (Dharshini and Mandal, 2025). We also visualized the DEGs using GEO2R interactive web tool and presented volcano plots for different datasets.

### 2.4 Identification of target genes for the alkaloids

To find out the potential ovarian cancer gene targets of the major alkaloids (Piperine, Piperlongumine, Piperlonguminine) of *P. longum,* we used Swiss target prediction (https://www.swisstargetprediction.ch/, accessed on 5 February 2026), GeneCards (https://www.genecards.org/, accessed on 19 February 2026) and comparative toxicogenomics database (CTD) (https://ctdbase.org/) by providing the SMILES for each alkaloid procured from PubChem database (https://pubchem.ncbi.nlm.nih.gov/) (Kanwal et al., 2022; Singh et al., 2023; Stelzer et al., 2016; Davis et al., 2025).

### 2.5 Screening of shared targets of alkaloids and ovarian cancer DEGs

The shared targets between the “upregulated DEGs vs. alkaloids targets” and “downregulated DEGs vs. alkaloids targets” were identified through a web tool called Venny 2.1.0 (https://bioinfogp.cnb.csic.es/tools/venny/) by entering the alkaloids targets and disease genes in the query (Oliveros, 2007).

### 2.6 Construction of protein-protein interaction (PPI) network

The interaction among the identified DEGs was performed using the STRING database v12.0 (https://string-db.org/) to investigate the potential functional interaction among proteins (Von Mering et al., 2005; Szklarczyk et al., 2023). The predicted protein-protein interactions of the target genes were obtained at the STRING database based on the medium confidence score of 0.4 or more as outlined by the STRING probabilistic score system. Confidence score of a given predicted interaction reflects the probability that this reported interaction will represent a real biological association and is obtained through combined evidence, e.g., experimental evidence, co-expression, curated databases and text mining. A cutoff of 0.4 or above was chosen to provide the best trade-off between network connectivity and specificity since cutoffs greater than this (≥0.7) disaggregated the PPI network and removed potentially significant interactions.

### 2.7 Visualization of protein interaction network and identification of key genes

The protein interaction network (PIN) obtained from STRING database was imported and visualized using Cytoscape 3.10.3 (https://cytoscape.org/). Topological analysis of the network was performed using the CytoHubba plugin to identify the top 10 hub genes based on six centrality algorithms, including maximal clique centrality (MCC), maximum neighbourhood component (MNC), degree, closeness, betweenness, and stress centralities (Chin et al., 2014). The ranks generated by the six algorithms for each gene were combined to compute an average rank, and the genes with highest overall scores were identified as hub genes. Gene clusters that were closely connected within the protein interaction network (PIN) were identified using the Molecular Complex Detection (MCODE) plugin. The core targets obtained from CytoHubba were then compared with MCODE clusters with scores greater than 3, using default parameters, to further refine the identification of biologically relevant genes (Shannon et al., 2003).

### 2.8 Functional and pathway enrichment analysis based on gene ontology

The biological functions and pathway enrichment associated with the key genes were identified using ShinyGO v0.85 (https://bioinformatics.sdstate.edu/go/). Statistical significance was determined by applying the Benjamini–Hochberg false discovery rate (FDR) adjustment with a significant threshold of 0.05 (Wang et al., 2025). Subsequently, the top 10 enriched Gene Ontology (GO), including biological process (BP), molecular function (MF) and cellular component (CC), and Kyoto Encyclopedia of Genes and Genomes (KEG) pathways were selected. The values of the fold enrichment for each term were taken directly from the ShinyGO enrichment output. Fold enrichment represents the extent of the enrichment of genes in a particular pathway and is calculated as the ratio of observed to expected overlap (k/E) where k represents the number of submitted genes identified in a particular GO/KEGG term, and E represents the expected overlap. The expect overlap was calculated using the formula $E=\frac{K}{N}xn$ , where n is the number of the input genes (10 genes) and K is the number of genes annotated to that particular term, while N is the background gene universe automatically selected by the enrichment analysis tool.

### 2.9 Analysis of gene expression and patient survival

The gene expression levels between ovarian cancer vs. normal ovarian tissues were analysed using the Gene Expression Profiling Interactive Analysis 2 (GEPIA2) server (http://gepia2.cancer-pku.cn/) (Tang et al., 2017). Differential expression was determined using thresholds log2 FC ≥ 1.0 and p ≤ 0.05. Furthermore, the overall survival (OS) of ovarian cancer patients was evaluated using the Kaplan-Meier (KM) plotter (https://kmplot.com/analysis/).

### 2.10 Mutation analysis

The Gene set cancer analysis (GSCA) database (https://guolab.wchscu.cn/GSCA/) was used to analyze the mutation frequency. This database contains comprehensive information on genomic alterations, including single-nucleotide variants (SNVs), across different cancer types such as ovarian cancer. SNV data associated with the hub genes were obtained and examined to determine mutation frequency and the variant types identified in ovarian cancer samples.

### 2.11 Molecular docking

Molecular docking analysis was performed to evaluate the binding potential of ligands against different protein targets. Protein structures were obtained from Uniprot database (https://www.uniprot.org/) and was modelled using AlphaFold 2.0 server. Preprocessing included removal of non-essential heteroatoms such as crystallographic water molecules, ions, followed by addition of missing hydrogen atoms to ensure proper valency and protonation states. The structures were converted into AutoDock-compatible PDBQT format using AutoDockTools, during which atom types were set and Gasteiger partial charges were calculated to allow for accurate modelling of electrostatic interactions. Ligand preparation was done by adding hydrogens, assign rotatable bonds, and compute partial charges. Ligands were then converted into flexible PDBQT format. Probable binding sites on proteins were predicted using the fpocket program, which detects surface cavities based on geometric and physicochemical properties. Coordinates of the chosen pocket were obtained, and the docking pocket space was determined by determining the geometric centre and the size of the pocket atoms, followed by adding a padding to the dimensions of the pocket to ensure that the ligand sampling is fully contained.

The docking was performed using AutoDock Vina (version 1.2.0) (Eberhardt et al., 2021), which uses a hybrid empirical scoring function and a stochastic global optimizer. In the docking process, a series of ligand poses are generated in the grid box (receptor is assumed to be rigid) and scored using the scoring function to rank the binding affinity. The Vina scoring function estimates the free energy of binding as a weighted sum of terms that account for steric complementarity, hydrophobic interactions, hydrogen bonding, lost degrees of freedom (conformational entropy) associated with the flexibility of the ligand, and non-bonded interaction potentials (distance-dependent functions). Instead of estimating thermodynamic free energies, the scoring function gives relative binding affinities (in kcal/mol) that can be used to rank poses based on their relative stability within the pocket. The ligands were docked with each protein receptor with the same docking parameters, such as exhaustiveness levels and multiple binding poses per complex. The predicted binding energies and pose coordinates were recorded and the lowest predicted binding energy located within the predicted pocket region was chosen as the representative pose. The protein-ligand complex interaction was shown in 2D interactions were generated using BioVia Discovery studio (Biovia DS, 2021).

### 2.12 Molecular dynamics simulation

Protein-ligand complexes were subjected to molecular dynamics (MD) simulations. These were carried out with the CHARMM force field in the GROMACS package (version 2023.1). The initial structure for each protein-ligand complex was obtained from docking simulations. The CHARMM-GUI server was used to generate the force field parameters. The CHARMM36m force field was employed to govern the protein dynamics, while the topologies and parameters for the individual alkaloid ligands were specifically generated using the CHARMM General Force Field (CGenFF) program (version 2.5.1) to ensure validated, highly accurate representation of small organic molecules. The structures were solvated with the TIP3 solvent model, Monte-Carlo ion placement method, and Na+ or Cl-counterions to balance the charge. All the simulations were energy minimized using the steepest descent method, and then equilibrated for 1 ns, with each structure heated from 100 K to 310.15 K. The equilibrated complexes were then subjected to 200 ns productive molecular dynamics run in the NPT ensemble at 310.15 K and 1 atm pressure. The velocity-rescale algorithm was used to control temperature and the Berendsen barostat was used to control pressure. The Particle-Mesh-Ewald (PME) method was applied to implement the periodic boundary condition for the explicitly solvated systems, and a 1-nm cut off distance was used for short-range interactions The covalent bonds containing hydrogen atoms were constrained using the P-LINCS algorithm 2-fs timestep was used. Once the simulations were finished, water molecules, translational and rotational motions were removed from the trajectory. Only the internal motions of the protein-ligand complex were analysed. In each simulation, the root means square deviation (RMSD) was calculated to track the overall conformational changes of the protein and the ligand. The root means square fluctuations (RMSF) of each residue was also calculated to evaluate the local flexibility and rigidity of the protein upon ligand binding.

## 3 Results

### 3.1 Pharmacokinetic profiling and drug-likeliness assessment

The *P. longum* alkaloids (piperine, piperlongumine, and piperlonguminine) have been profiled to assess their physiochemical and pharmacokinetic properties to predict their drug-likeness and oral bioavailability. The three alkaloids strictly followed to Lipinski’s rule of five with no violation (As shown in Table 1), which is a clear indication of their potential as oral drugs. The molecular weights of all the three compounds were significantly lower than 500 g/mol threshold, and the lipophilicity (Consensus LogP) values (1.96–3.17) were in an ideal range for efficient permeation through cellular membranes. Moreover, the topological polar surface area (TPSA) values were low (38.77–65.07 Å^2^), and this is well aligned with the computational predictions of high gastro-intestinal (GI) absorption and high active blood-brain barrier permeability for the three alkaloids. Therefore, these ADME profiles demonstrate that *P. longum* alkaloids possess excellent pharmacokinetic properties, and can be considered as potent and bioavailable chemotherapeutics for ovarian cancer treatment.

**TABLE 1:** Physiochemical properties, drug-likeness, and ADME profile of *P. longum* alkaloids and reference drug evaluated via SwissADME.

Compound	Molecular weight (g/mol)	H-bond donors	H-bond acceptors	TPSA (Å^2^)	LogP (consensus)	GI absorption	Blood-Brain-Barrier permeability	Lipinski violations	Bioavailability score
Piperine	285.34	0	3	38.77	3.03	High	Yes	0	0.55
Piperlongumine	317.54	0	5	65.07	1.96	High	Yes	0	0.55
Piperlonguminine	273.33	1	3	47.56	3.17	High	Yes	0	0.55
Paclitaxel (Reference drug)	853.91	4	14	221.29	3.95	Low	No	2	0.17

The table highlights predicted gastrointestinal (GI) absorption, blood-brain barrier (BBB) permeability, and bioavailability scores. Paclitaxel is included as a standard reference drug for comparison. Compliance with Lipinski’s rule of five includes the oral bioavailability potential of the compounds.

### 3.2 Toxicity and safety profiling of *Piper longum* alkaloids

The overall toxicity profile of the three individual *P. longum* alkaloids was also found to be very favourable when assessed *in silico* using a computational simulation platform called ProTox-3.0, indicating their potential as drug candidates. The *in silico* predictions indicated an absence of organ-specific damage, as piperine, piperlongumine, and piperlonguminine were all safely classified as inactive for hepatotoxicity, demonstrating strong prediction probabilities of 0.91, 0.79, and 0.80, respectively (as shown in Table 2). The predictions for cardiotoxicity also showed that it was safe, with probabilities of 0.77, 0.59 and 0.50 for piperine, piperlongumine, and piperlonguminine, respectively. In addition, the compounds showed high genetic safety, with no showing mutagenic potential, and piperine, piperlongumine and piperlonguminine were predicted to be strictly inactive for mutagenicity with a probability of 0.96, 0.69, and 0.64, respectively. Concerning carcinogenicity, piperlongumine and piperlonguminine were also safely inactive with probabilities of 0.52 and 0.53, respectively, and piperine was labelled active, but its prediction probability was very close to the boundary (0.53), indicating a very low overall risk. Finally, piperine, piperlongumine and piperlonguminine are predicted for activity in immunotoxicity with probabilities of 0.96, 0.99, and 0.98, respectively, a property common to plant derived immunomodulatory agents in cancer therapy, while they exhibit no hepatotoxic, cardiotoxic and mutagenic activity, suggesting that these compounds have a safe and well-tolerated pharmacological profile for further drug development.

**TABLE 2:** Computational toxicity profiling of *P. longum* alkaloids.

Endpoint	Compound	Prediction status	Probability score
Hepatotoxicity	Piperine	Inactive	0.91
	Piperlongumine	Inactive	0.79
	Piperlonguminine	Inactive	0.80
Cardiotoxicity	Piperine	Inactive	0.77
	Piperlongumine	Inactive	0.59
	Piperlonguminine	Inactive	0.50
Carcinogenicity	Piperine	Active	0.53
	Piperlongumine	Inactive	0.52
	Piperlonguminine	Inactive	0.53
Immunotoxicity	Piperine	Active	0.96
	Piperlongumine	Active	0.99
	Piperlonguminine	Active	0.98
Mutagenicity	Piperine	Inactive	0.96
	Piperlongumine	Inactive	0.69
	Piperlonguminine	Inactive	0.64

“Active” indicates a predicted risk threshold breach by the model, whereas “Inactive” implies a safe computational profile for that specific toxicological assay. The probability scores represent the model's prediction confidence level.

### 3.3 Screening of DEGs

Gene Expression Omni bus (GEO) database was used to obtain ovarian cancer specific transcriptomic data base. Then datasets were pre-processed with the help of GEO2R interactive web tool (https://www.ncbi.nlm.nih.gov/geo/geo2r/). At first there were 14,063 DEGs altogether from the five datasets (GSE10971, GSE12470, GSE26712, GSE29450, and GSE54388) which were collected from Gene Expression Omni bus (GEO) database. Then these were refined for log2 FC and adj. P value after which we got a list of 5,836 upregulated and 8,227 downregulated genes in the datasets.

**FIGURE 1:**
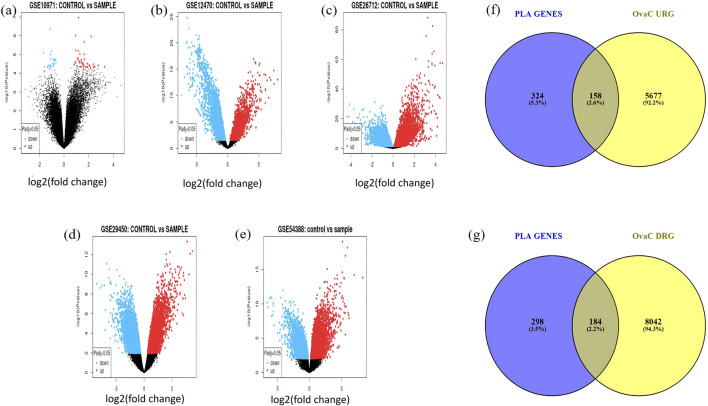
**(a–e)** Volcano plots of Differentially Expressed Genes (DEGs) across ovarian cancer datasets and Venn diagrams of overlapping genes depicting **(f)** *Piper longum* alkaloids (PLA) target genes vs. upregulated genes in ovarian cancer (OvaC URG), **(g)** *Piper longum* alkaloids target genes (PLA) vs. downregulated genes in ovarian cancer (OvaC DRG).

The biological significance of these DEGs were assessed through functional annotation using g:profiler. Expression of these DEGs were visualized through volcano plots in which red dots were denoting upregulated genes and blue dots represent downregulated genes, thereby allowing us to view the gene expression pattern. A total of 483 *P. longum* alkaloids target genes were obtained from comparative toxicogenics database (CTD), GeneCards, and Swiss target prediction. To obtain the most reliable data in the gene set, duplicates were removed. Venny 2.1.0 was used to study the overlap between *P. longum* alkaloids target genes and ovarian cancer related genes, which revealed an overlap 342 genes, comprising 158 upregulated genes and 184 downregulated genes (Figure 1). These are all differentially expressed genes in ovarian cancer and expected to have speculative or confirmed binding with *P. longum* alkaloids. These are functionally relevant *P. longum* alkaloids’ target genes in ovarian cancer.

### 3.4 Identification of hub genes using cytoHubba and MCODE plugins

**FIGURE 2:**
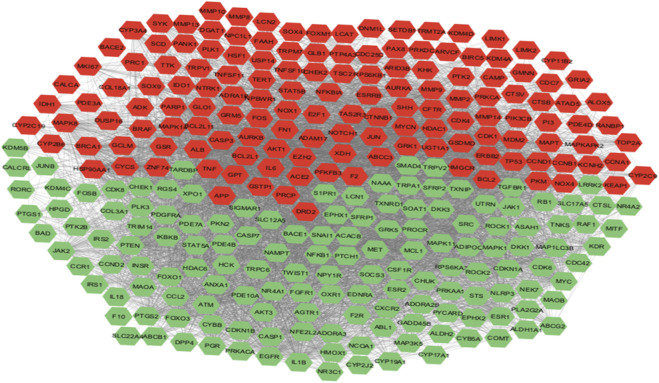
Visualization of PPI network using Cytoscape. Hexagonal nodes represent individual genes, and connecting lines (edges) indicate interactions between them. Node colour designates status: green indicates upregulated genes (158), and red indicates downregulated genes (184).

**FIGURE 3:**
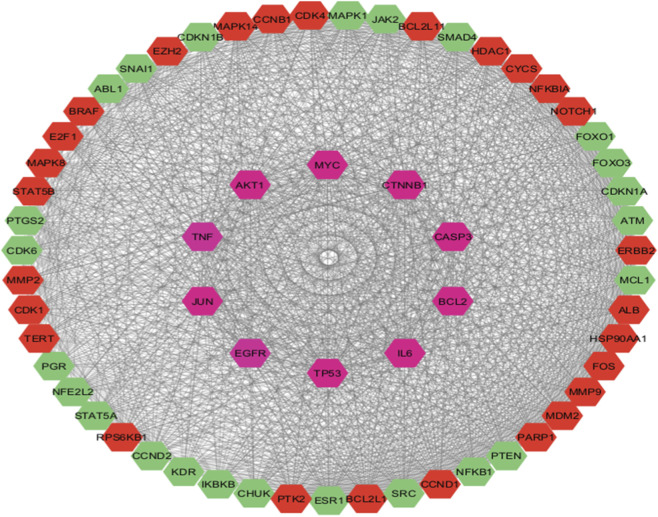
Gene clusters identified using MCODE, with hub genes highlighted in purple hexagons in the centre: Cluster 1 (score: 52.6). Clusters 2, 3, 4, five did not include any of the identified hub genes.

The Protein-protein interaction (PPI) network of the DEGs with a confidence score threshold of ≥0.4 were obtained from the STRING database which consisted 292 nodes and 6,373 edges. This network had an average node degree of 43.7 and an average local clustering coefficient of 0.595. PPI enrichment analysis was done to calculate a highly significant p-value of 1.0 × 10^−16^ (p < 0.05), which indicated that these DEGs show more interactions than expected for a random set of proteins of same size, suggesting a strong response with coordination (Figure 2). The cytoHubba plugin was used to identify the top 10 hub genes associated with ovarian cancer survival and progression based on rankings derived from six topological parameters. In addition, the MCODE plugin was applied to detect significant gene clusters within the PPI network. The clusters identified were subsequently compared with the hub genes obtained (Figure 3). As shown in Table 3, The genes TP53, CTNNB1, AKT1, IL6, TNF, EGFR, CASP3, BCL2, MYC and JUN constantly appeared across several rankings in cytoHubba and were more concentrated within clusters with scores >3.

**TABLE 3:** Top 10 hub genes identified using cytoHubba based on six topological metrics.

S. no	MCC	MNC	Degree	Closeness	Betweenness	Stress
1	TP53*	TP53*	TP53*	TP53*	AKT1*	AKT1*
2	BCL2*	AKT1*	AKT1*	AKT1*	TP53*	TP53*
3	CASP3*	TNF*	TNF*	TNF*	SRC	TNF*
4	CTNNB1*	IL6*	IL6*	IL6*	EGFR*	IL6*
5	MYC*	BCL2*	BCL2*	BCL2*	CTNNB1*	SRC
6	PTEN	CTNNB1*	CTNNB1*	CTNNB1*	TNF*	EGFR*
7	CCND1	EGFR*	EGFR*	EGFR*	IL1B	IL1B
8	AKT1*	CASP3*	CASP3*	CASP3*	IL6*	ALB
9	IL6*	MYC*	MYC*	MYC*	ALB	CTNNB1*
10	NFKB1	JUN*	JUN*	JUN*	ESR1	ESR1

Among the 12 topological parameters in cytoHubba, MCC, MNC, Degree, Closeness, Betweenness, and Stress were used to identify the key hub genes (repetition ≥2). These genes were further validated by cross-referencing with gene clusters having an MCODE score >3. Genes marked with * denote the key hub genes identified through this integrative approach.

### 3.5 Gene ontology (GO) and KEGG functional enrichment analysis

Gene ontology (GO) enrichment analysis was carried out using ShinyGO v0.85 to assess the biological functions of the ten key P. longum alkaloid genes in ovarian cancer. The GO biological process (BP) enrichment analysis revealed 172 significantly enriched pathways, with top enriched terms including downregulation of apoptotic process, response to xenobiotic stimulus, cell population proliferation, upregulation of apoptotic process and positive regulation of transcription by RNA polymerase-II. A total of 20 enriched pathways were identified in the Cellular component (CC) catergory. The encoded proteins are predominantly localized within the nucleus and are highly organized into broader protein-containing complexes. Importantly, sub-cellular localization indicates a strong concentration within functionally active nuclear compartments, most notably euchromatin, which exhibited a substantial fold enrichment of 97.54 (p = 3.31 × 10^−4^). The extreme fold enrichment (97.54) for euchromatin localization is an artifact of the small input size (n = 10) against a low background count (0.03). However, the significant p-value (p = 3.31 × 10^−4^) validates that this overrepresentation of master regulatory genes is biologically genuine.

In addition, the targets were significantly enriched for transcription regulator complexes and the ciliary basal body. Enrichment analysis of Molecular function (MF) identified 23 significantly enriched pathways, which indicates that the target genes are significantly involved in complex protein-protein interactions and regulation of the enzymatic pathways. The top significant molecular function was ubiquitin protein ligase binding, suggesting a key role in regulating protein stability and degradation. The encoded protein also showed a high degree of identical protein binding and general enzyme binding, including proteinase binding, kinase binding, and protein phosphatase binding. Consistent with their nuclear localization, the targets also showed strong chromatin binding and DNA-binding transcription factor binding. Most importantly, the overall GO pathway enrichment analysis showed that the target genes are primarily associated with regulation of apoptosis, cell proliferation and response to xenobiotic stimuli, highlighting the significance of these hub genes as key transcriptional and enzymatic regulators in regulating cancer cell survival and the pharmacology of individual alkaloid treatment. The top 10 pathways of all the categories are listed down in Table 4.

**TABLE 4:** Gene ontology (GO) analysis.

Biological process			
Pathways	No. of genes	Gene ID	Fold enrichment
Positive regulation of miRNA transcription	06	IL6, JUN, MYC, TNF, TP53, EGFR	191.92
Response to glucocorticoid	04	IL6, CASP3, BCL2, TNF	144.53
Response to xenobiotic stimulus	07	JUN, MYC, CASP3, BCL2, CTNNB, TNF, TP53	52.3
Cell population proliferation	07	JUN, MYC, BCL2, CTNNB1, AKT1, TP53, EGFR	51.16
Positive regulation of apoptotic process	07	IL6, JUN, CASP3, BCL2, CTNNB1, TNF, TP53	47.82
Regulation of cell population proliferation	05	JUN, CTNNB1, TNF, TP53, EGFR	41.02
Negative regulation of apoptotic process	09	IL6, JUN, MYC, BCL2, CTNNB1, AKT1, TNF, TP53, EGFR	32.34
Negative regulation of cell population proliferation	06	IL6, JUN, BCL2, CTNNB1, TNF, TP53	26.02
Positive regulation of gene expression	06	IL6, MYC, CTNNB1, AKT1, TNF, TP53	22.73
Positive regulation of transcription by RNA polymerase II	08	IL6, JUN, MYC, CTNNB1, AKT1, TNF, TP53, EGFR	12.56
Cellular component			
Euchromatin	03	JUN, MYC, CTNNB1	97.54
Transcription repressor complex			64.02
Transcription regulator complex	03	JUN, CTNNB1, TP53	24.87
Protein-containing complex	07	MYC, BCL2, CTNNB1, AKT1, TNF, TP53, EGFR	18.28
Ciliary basal body	03	CTNNB1, AKT1, EGFR	18.20
Glutamatergic synapse	03	CASP3, CTNNB1, AKT1	10.51
Cilium	03	CTNNB1, AKT1, EGFR	9.37
Endoplasmic reticulum	04	BCL2, AKT1, TP53, EGFR	4.73
Nucleoplasm	06	JUN, MYC, CASP3, CTNNB1, AKT1, TP53	3.05
Nucleus	08	JUN, MYC, CASP3, BCL2, CTNNB1, AKT1, TP53, EGFR	2.36
Molecular function			
General transcription initiation factor binding	02	JUN, TP53	321.2
Protease binding	04	CASP3, BCL2, TNF, TNF, TP53	68.22
Protein phosphatase binding	03	BCL2, CTNNB1, EGFR	60.23
Kinase binding	03	CTNNB1, AKT1, EGFR	54.54
Ubiquitin protein ligase binding	06	JUN, MYC, BCL2, CTNNB1, TP53, EGFR	37.91
DNA-binding transcription factor binding	03	MYC, BCL2, CTNNB1	31.25
RNA polymerase II-specific DNA-binding transcription factor binding	03	JUN, CTNNB1, TP53	29.5
Enzyme binding	05	JUN, CTNNB1, AKT1, TP53, EGFR	22.94
Chromatin binding	04	JUN, CTNNB1, TP53, EGFR	14.71
Identical protein binding	08	IL6, JUN, MYC, BCL2, AKT1, TNF, TP53, EGFR	8.45

Based on the KEGG pathway enrichment analysis, a total of 20 distinct signalling cascades were identified, with ten most highly significant pathways outlined in Table 5. The identifies hub genes demonstrated prominent involvement in colorectal cancer, followed closely by endometrial cancer, thyroid cancer, bladder cancer, and anti-folate resistance. The strong association of these target genes with diverse malignancies and specific resistance mechanisms (endocrine and platinum drug resistance) strongly indicates that the *P. longum* alkaloids may exert its anti-tumour efficacy by extensively rewiring core oncogenic networks and overcoming mechanisms of chemoresistance.

**TABLE 5:** Top 10 enriched pathways of the hub genes.

S. no	KEGG pathways	No. of genes	Gene ID	Fold enrichment
1.	Colorectal cancer	08	CTNNB1, EGFR, AKT1, JUN, MYC, BCL2, TP53, CASP3	213.8667
2.	Endometrial cancer	05	CTNNB1, EGFR, AKT1, MYC, TP53	197.1017
3.	Thyroid cancer	03	CTNNB1, MYC TP53	188.5784
4.	Bladder cancer	03	EGFR, MYC, TP53	170.1805
5.	Antifolate resistance	02	IL6, TNF	155.0533
6.	Apoptosis-multiple species	02	BCL2, CASP3	145.3625
7.	AGE-RAGE signalling pathway in diabetic complications	06	AKT1, IL6, JUN, BCL2, TNF, CASP3	140.9576
8.	Central carbon metabolism in cancer	04	EGFR, AKT1, MYC, TP53	131.031
9.	African trypanosomiasis	02	IL6, TNF	129.2111
10.	Small cell lung cancer	05	AKT1, MYC, BCL2, TP53, CASP3	125.043

### 3.6 Validation of hub gene expression in ovarian cancer

**FIGURE 4:**
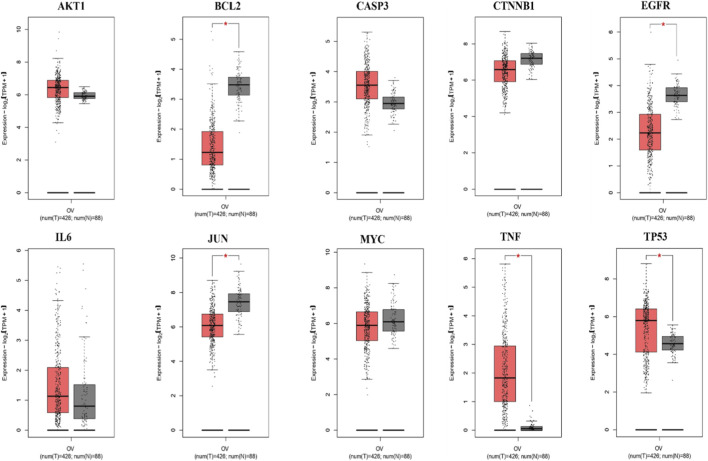
Expression levels of core hub genes in ovarian cancer. Box plots comparing the expression of the top 10 identified hub genes between ovarian tumour tissues (red boxes, n = 426) and normal tissues (grey boxes, n = 88) (* indicates p < 0.05), OV (ovarian cancer).

The expression levels of the hub genes were analyzed using GEPIA2 to compare their expression between normal and tumour samples. The expression data were represented as log_2_ (TPM+1) for normalization. Among these, five genes, namely, BCL2, EGFR, JUN, TNF and TP53 exhibited significantly altered expression profiles in the tumour samples compared to normal controls (p ≤ 0.05). Contrarily, the remaining genes (AKT1, CASP3, CTNNB1, IL6 and MYC) did not show any differences that are statistically significant. Which indicates relatively stable baseline expression between the normal and tumour tissues (Figure 4).

### 3.7 Prognostic analysis of overall survival

**FIGURE 5:**
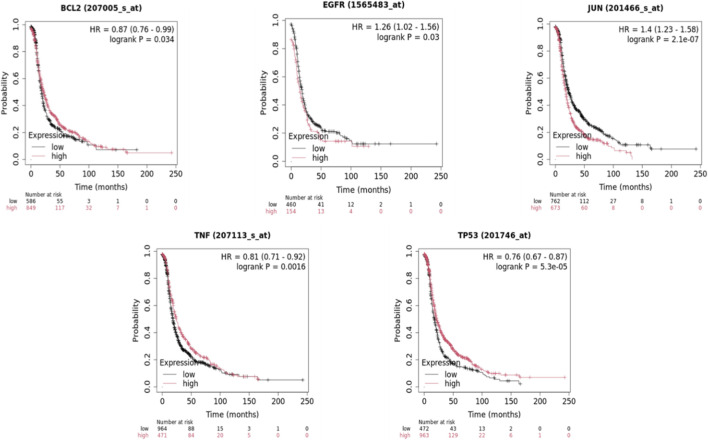
Kaplan-Meier survival analysis of core hub genes. Overall survival curves of ovarian cancer patients stratified by high (red) and low (black) expression levels of BCL2, EGFR, JUN, TNF, and TP53. P < 0.05 and HR > 1 (hazard ratio) considered statistically significant.

The predictive significance of five crucial upregulated hub genes, namely, BCL2, EGFR, JUN, TNF and TP53, were evaluated for overall survival in ovarian cancer patients using Kaplan-Meier (KM) plotter. Among these five genes, JUN exhibited the strongest adverse prognostic impact with a hazard ratio (HR) of 1.4 (confidence interval (CI): 1.23-1.58; p = 2.1e-07), indicating that higher expression levels were associated with poorer survival outcomes. This was followed by EGFR (HR = 1.26, CI: 1.02-1.56, p = 0.03), which was also associated with reduced survival probability. Followed by other hub genes TP53 (HR = 0.76, CI: 0.76-0.87, p = 5.3e-05), followed by TNF (HR = 0.81, CI: 0.71-0.92, p = 0.0016) and BCL2 (HR = 0.87, CI: 0.76-0.99, p = 0.034) (Figure 5).

### 3.8 Analysis of mutations in hub genes

The Gene set cancer analysis (GSCA) database (https://guolab.wchscu.cn/GSCA/) was used to analyze the mutation frequency. This analysis utilized genomic data from 374 samples belonging to the TCGA-OV (The Cancer Genome Atlas - Ovarian Serous Cystadenocarcinoma) cohort. Within the GSCA platform, mutation calling is based on the standardized Genomic Data Commons (GDC) pipeline, which aggregates and processes somatic variants. Mutation profiling of the hub genes revealed that missense mutations were the most predominant variant classification, followed by nonsense mutations and frame-shift deletions. In terms of the variants, the majority of genetic changes were single nucleotide polymorphisms (SNPs), whereas insertions and deletions were rare. When the single nucleotide variants (SNVs) were examined, C > T was the most common mutation (153 occurrences), followed by T > C (55), C > A (51), C > G (23), T > A (18), and T > G (18). The median number of variants per sample was one, suggesting a low mutation burden among the examined genes. TP53 was found to be the most commonly mutated gene (100%) among the genes analysed (representing the High-Grade Serous Ovarian Carcinoma subtype derived from the TCGA-OV cohort, processed *via* the GDC variant calling pipeline), followed by EGFR (2%), TNF (1%), MYC (1%), and JUN (1%). On the other hand, CASP3 and CTNNB1 genes were not found to have any mutations in the samples.

**FIGURE 6:**
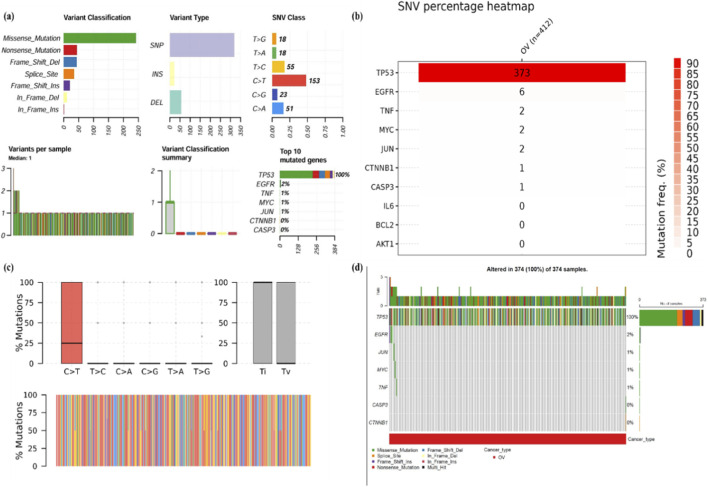
Somatic mutation profiling and genetic alteration landscape of the identified hub genes. The multi-panel figure illustrates the mutational characteristics of the top 10 hub genes within the ovarian cancer cohort. **(a)** Statistical summary of the mutations reveals that missense mutations and single nucleotide polymorphisms (SNPs) are the predominant variant classifications. **(b)** A heat map showing the absolute count and percentage of SNVs. **(c)** Boxplot and stacked bar charts detailing the distribution of transition (Ti) and transversion (Tv) mutation across the samples. **(d)** An oncoplot (waterfall plot) displaying the mutation distribution across 374 tumour samples.

The analysis of 374 ovarian cancer samples revealed that all samples have mutations in atleast one of the genes, with the majority of mutations occurring in TP53. The high frequency of mutation in TP53 gene and the mutations in other genes suggests their possible role in tumorigenesis and progression in ovarian cancer (Figure 6).

### 3.9 Molecular docking analysis

**FIGURE 7:**
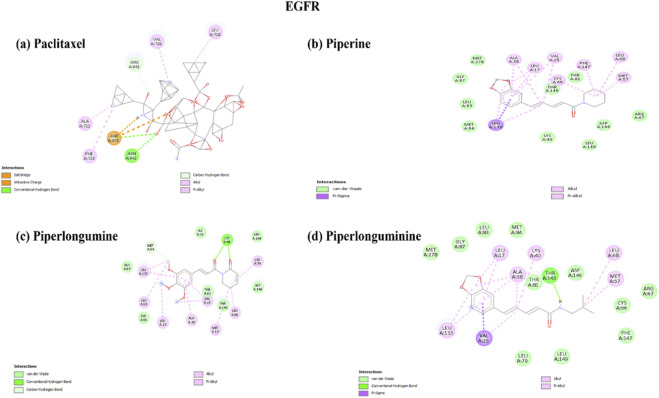
2D interaction profiles of Paclitaxel **(a)**, Piperine **(b)**, Piperlongumine **(c)**, and Piperlonguminine **(d)** with EGFR.

**FIGURE 8:**
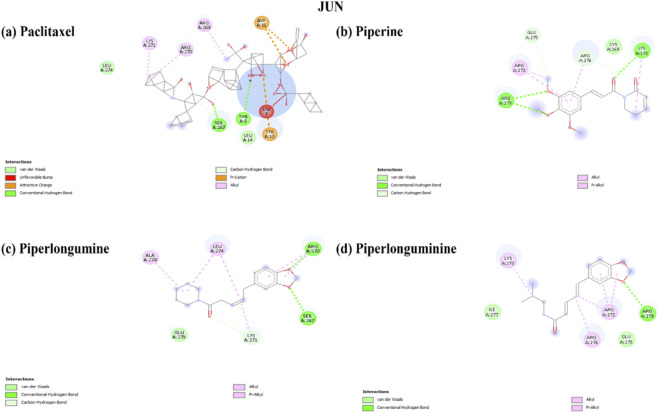
2D interaction profiles of Paclitaxel **(a)**, Piperine **(b)**, Piperlongumine **(c)**, and Piperlonguminine **(d)** with JUN.

**FIGURE 9:**
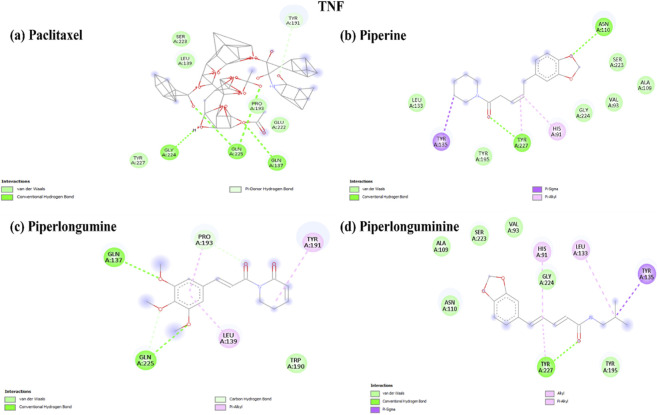
2D interaction profiles of Paclitaxel **(a)**, Piperine **(b)**, Piperlongumine **(c)**, and Piperlonguminine **(d)** with TNF.

**FIGURE 10:**
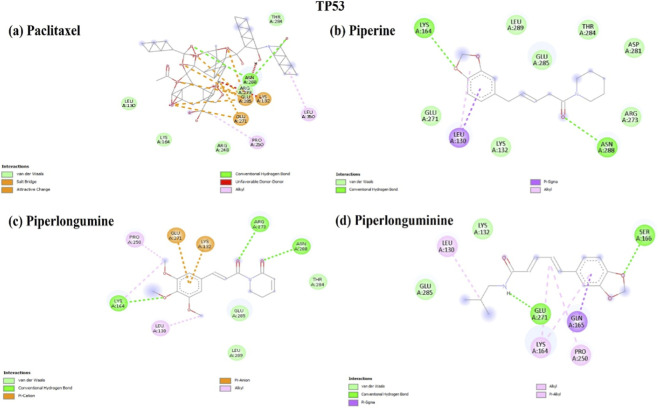
2D interaction profiles of Paclitaxel **(a)**, Piperine **(b)**, Piperlongumine **(c)**, and Piperlonguminine **(d)** with TP53.

Among the ligands evaluated, paclitaxel (reference drug) consistently exhibited the strongest binding affinity across all target proteins. The most significant interaction was observed between paclitaxel and EGFR, which yielded a binding energy of −12.47 kcal/mol. This was followed by interactions with TP53 (−7.63 kcal/mol) and TNF (−7.60 kcal/mol). JUN showed the weakest binding for paclitaxel at −6.65 kcal/mol. Overall, the binding hierarchy for paclitaxel across the target proteins was EGFR > TP53 > TNF > JUN, suggesting that EGFR provides the most stable and complimentary binding pocket for this drug. Totally it formed 10 hydrogen bonds across the four targets (EGFR-01, JUN-03, TNF-03, TP53-03). Among the evaluated alkaloids, Piperlongumine formed the same number of hydrogen bonds as paclitaxel for three out of four targets (EGFR-01, JUN-03, and TP53-3). It also exhibited the highest number of hydrogen bonds (09) among the alkaloids followed by Piperlonguminine (07), and Piperine (05). Piperine demonstrated the highest binding affinity, interacting most strongly with EGFR (−8.70 kcal/mol). This was followed by Piperlonguminine (−8.45 kcal/mol) and Piperlongumine (−8.22 kcal/mol) for the same protein target. A similar trend was observed across the other proteins (TNF, TP53, and JUN), where the alkaloids consistently showed moderate affinities ranging from −5.01 to −5.71 kcal/mol. The Table 6; Figures 7–10 gives a complete summary of the docking results.

**TABLE 6:** Molecular docking interactions of *P. longum* alkaloids and paclitaxel (reference drug) against key hub genes.

Ligand	Name of the gene	PBD ID	Binding affinity (kcal/mol)	No. of hydrogen bonds	Interacting residues
Piperine	EGFR	1XKK	−8.70	01	Met 278, Gly 87, Leu 83, Met 84, Leu 135, Lys 40, Leu 149, Asp 146, Arg 67, Met 57, Leu 68, Phe 147, Thr 81, Cys 66, Thr 145, Val 25, Leu 17, Ala 38
	JUN	P05412	−5.17	01	Ala 278, Leu 274, Arg 270, Ser 267, Lys 271, Glu 275
	TNF	P01375	−5.71	01	Leu 133, Tyr 135, Tyr 227, His 91, Gly 224, Val 93, Ala 109, Ser 223, Asn 110
	TP53	P04637	−5.53	02	Glu 271, Lys 164, Leu 289, Glu 285, Thr 284, Asp 281, Arg 273, Asn 288, Lys 132, Leu 130,
Piperlongumine	EGFR	1XKK	−8.22	01	Met 84, Gly 87, Leu 135, Leu 83, Cys 88, Leu 17, Ala 38, Val 25, Thr 81, Met 57, Thr 145, Leu 68, Asp 146, Leu 79, Leu 149, Lys 40, Ile 39
	JUN	P05412	−5.08	03	Arg 279, Arg 272, Glu 275, Arg 276, Cys 269, Lys 273
	TNF	P01375	−5.15	02	Gln 137, Pro 193, Tyr 191, Trp 190, Leu 139, Gln 225,
	TP53	P04637	−5.11	03	Lys 164, Pro 250, Glu 271, Lys 132, Arg 273, Asn 273, Asn 288, Thr 284, Glu 285, Leu 289, Leu 130,
Piperlonguminine	EGFR	1XKK	−8.45	01	Leu 135, Val 25, Leu 79, Leu149, Phe 147, Cys 66, Arg 67, Met 57, Leu 68, Asp 146, Thr 145, Thr 81, Ala 38, Lys 40, Leu 17, Met 84, Leu 83, Gly 87, Met 278
	JUN	P05412	−5.22	03	Lys 273, Ile 277, Arg 276, Arg 272, Glu275, Arg 279
	TNF	P01375	−5.42	01	Asn 110, Ala 109, Ser 223, Val 93, His 91, Gly 224, Leu 133, Tyr 135, Tyr 195, Tyr 227, Asn 110
	TP53	P04637	−5.01	02	Glu 285, Leu 130, Lys 132, Ser 166, Glu 271, Gln 165, Lys 164, Pro 250
Paclitaxel (Reference drug)	EGFR	1XKK	−12.47	01	Ala 722, Phe 723, Asp 855, Asn 842, Leu 718, Val 726, Arg 841
	JUN	P05412	−6.65	03	Leu 274, Lys 271, Arg 270, Arg 263, Asp 11, Tyr 10, Lig 1, Thr 8, Leu 14, Ser 267
	TNF	P01375	−7.60	03	Ser 223, Leu139, Tyr 227, Gly 224, Gln 225, Gln 137, Glu 222, Pro 193, Tyr 191
	TP53	P04637	−7.63	03	Leu 130, Lys 164, Arg 248, Pro 250, Glu 271, Glu 285, Arg 273, Asn 288,

### 3.10 Molecular dynamics simulation

Molecular dynamics simulations over 200 ns utilizing GROMACS package (version 2023.1) with the Charmm force field. The structures were solvated with the TIP3 solvent model, Monte-Carlo ion placement method, and Na + or Cl-counterions to balance the charge. All the simulations were energy minimized using the steepest descent method, and then equilibrated for 1 ns, with each structure heated from 100 K to 310.15 K. The equilibrated complexes were then subjected to 200 ns productive molecular dynamics run in the NPT ensemble at 310.15 K and 1 atm pressure.

Following these parameters, the 200 ns production runs demonstrated the long-term stability of the four protein–ligand complexes, with no steady upward trend to suggest protein-ligand dissociation. EGFR–piperine was the most stable, rapidly reaching a plateau in a narrow 0.20–0.25 nm range. TP53–piperine and TNF–piperine showed some initial adjustments, then stabilized between 2.0–2.1 nm and 2.3–2.6 nm, respectively. JUN–piperlonguminine was the most flexible, reaching an equilibrium of 2.9–3.2 nm, which is in line with the flexible nature of JUN’s native structure.

Residue flexibility analysis showed that the residues forming the binding sites in all complexes were highly constrained, ensuring tight binding without distorting the overall structure. EGFR showed a highly constrained overall structure, with limited surface loop dynamics. TNF and TP53 maintained rigid cores with flexible termini and loops. JUN showed the largest backbone variations, consistent with its native regulatory motions.

Compactness and solvent accessibility (RG and SASA) analyses showed simultaneous compaction upon ligand binding. Although EGFR maintained a highly compact structure throughout the entire simulation, TNF, TP53, and JUN experienced rapid extreme compaction in the initial 10–40 ns. Such concerted stabilization enabled their hydrophobic cores to be buried, thereby considerably dampening initial solvent exposure, and preserving the folded integrity of the complexes.

**FIGURE 11:**
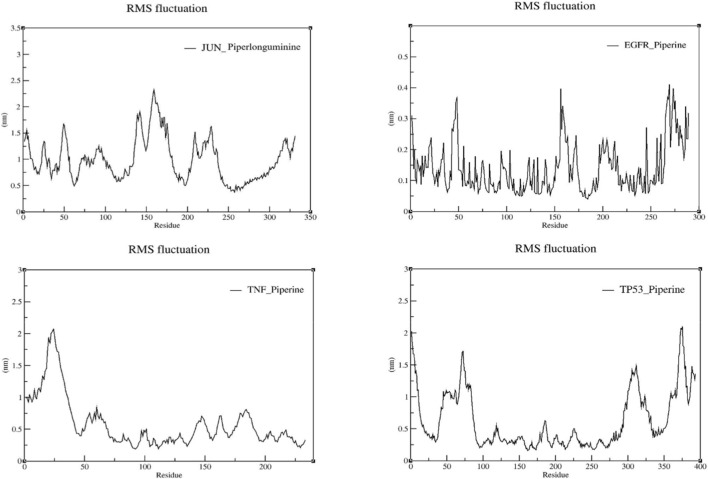
Root Mean Square Fluctuation (RMSF) analysis. RMSF values of protein residues calculated over a 200 ns MD simulation.

**FIGURE 12:**
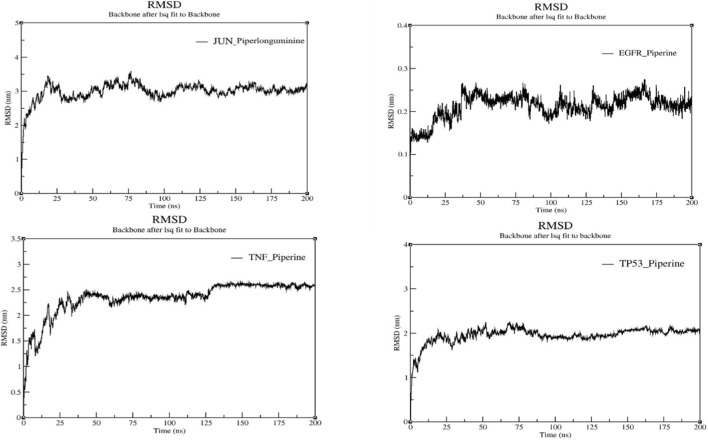
Root Mean Square Deviation (RMSD) analysis. Backbone RMSD trajectories over 200 ns.

**FIGURE 13:**
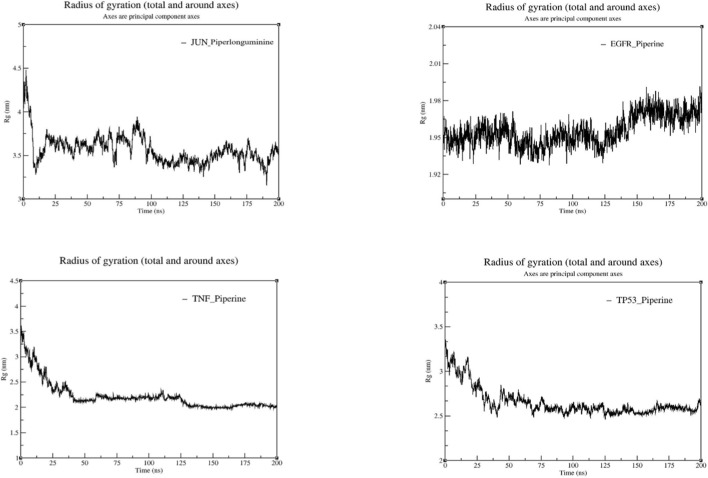
Radius of Gyration (Rg) analysis. Radius of gyration (Rg) plots over 200 ns.

**FIGURE 14:**
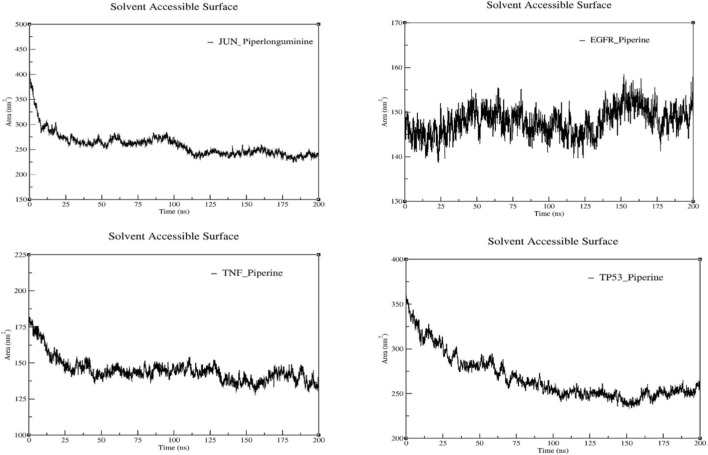
Solvent Accessible Surface Area (SASA) analysis. ASA evolution over 200 ns.

**FIGURE 15:**
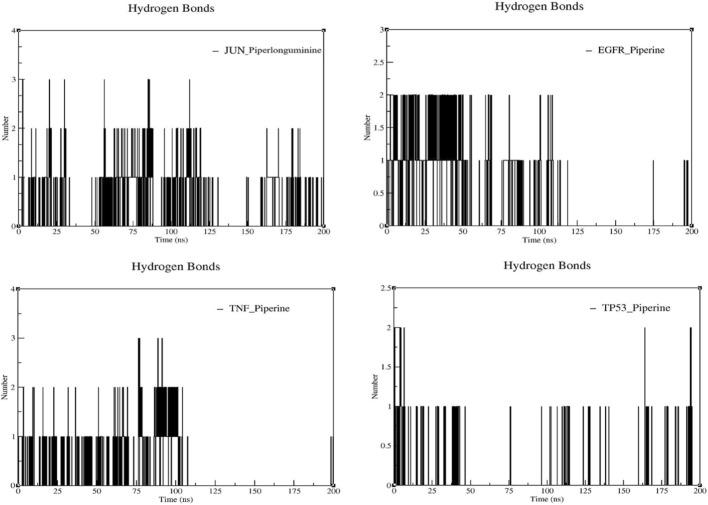
Intermolecular Hydrogen Bond analysis.

Overall, interaction dynamics suggested that the formation of the complex was driven by strong hydrophobic and van der Waals interactions, with occasional hydrogen bonding. EGFR and TP53 were heavily dependent on hydrophobic anchoring, and exhibited low, transient hydrogen bonding. TNF demonstrated moderate initial hydrogen bonding that evolved into stable non-polar interactions, whereas JUN had the most persistent hydrogen bonding, playing a key role in its initial positioning and long-term association. Figures 11–15 collectively illustrate the stability, conformational behaviour, and overall molecular dynamics simulation results. Taken together, the MD simulation findings corroborate the molecular docking results, reinforcing the binding specificity and therapeutic promise of piperine and piperlonguminine against their respective protein targets.

## 4 Discussion

Ovarian cancer remains a major clinical challenge, mainly due to late-stage diagnosis, frequent recurrence, and the emergence of chemoresistance, ovarian cancer continues to be one of the deadliest gynaecologic cancers. The exact regulatory networks that control the course of the disease are still unclear, despite the fact that a number of molecular changes have been linked to ovarian cancer parthenogenesis (Wang J et al., 2025). Radiation and chemotherapy can partially induce senescence inside the tumour mass. Chemotherapeutic drugs, however, have been shown to target both healthy and cancerous cells, which results in adverse effects. Several beneficial secondary metabolites have been found, particularly in the anti-tumor and anti-infection domains, and plants have been utilized to treat cancer since ancient times. Approximately 67% of the medications used in cancer chemotherapy are derived from natural ingredients (Zia-ur-Rehman et al., 2022).

Alkaloids are a potentially useful class of phytochemicals with anticancer effects. They are a class of basic organic compounds that are present in nature and of all of which include one or more nitrogen atoms (Singh et al., 2023). *Piper longum* is rich in alkaloids, which are known for their diverse pharmacological activities. Among these, piperine is the major amide alkaloid and has been widely reported for its multiple therapeutic effects, including neuroprotective, antihypertensive, anti-inflammatory, and anticancer properties. A second important compound, piperlongumine, has been shown to have selective toxicity to cancer cells and low overall toxicity, making it a promising candidate for the treatment of diseases such as psoriasis, Parkinson’s disease, and rheumatoid arthritis (Wan et al., 2023).

In this study, the ADME profiles of the *P. longum* alkaloids demonstrates that these compounds exhibit good drug-likeness and oral absorption, firmly establishing them as promising chemotherapeutic agents against ovarian cancer. As these alkaloids strictly adhered to Lipinski’s rule of five, exhibiting zero violations. Their molecular weights are well below the 500 g/mol threshold, and their lipophilicity indicates that they have optimal balance for cellular membrane penetration. Moreover, the Topological Polar Surface Area (TPSA) shows that they have high gastrointestinal absorption and active blood-brain barrier permeability. Collectively, these favourable physicochemical properties of *P. longum* alkaloids provide a rigorous computational foundation for their clinical translation.

The reliability of computational toxicity platforms like ProTox has been well-demonstrated across recent screening studies of plant-derived compounds. For instance, a virtual screening study for cervical cancer therapeutics utilized the platform to evaluate critical parameters like carcinogenicity and mutagenicity, emphasizing how *in silico* profiling accelerates the identification of low-toxicity leads while minimizing animal testing (Naveed et al., 2026).

The *in silico* toxicity prediction of *P. longum* alkaloids have been done using ProTox-3.0 prediction server. They have been shown to be highly promising as therapeutic agents without significant toxic side effects. They are not predicted to be hepatotoxic, cardiotoxic or mutagenic, suggesting a highly favourable and well-tolerated pharmacological baseline. In addition, although all three compounds are associated with immunotoxicity, this is often a sign of the beneficial immunomodulatory activity typical of many plant-based anti-cancer drugs, not immune suppression. The results are also largely negative for carcinogenicity, supporting the safety of these individual alkaloids, and offer a strong basis for further preclinical studies of these compounds.

The transcriptomic profiling with the help of GEO database and GEO2R web tool yielded 5,836 upregulated and 8,227 downregulated genes in ovarian cancer. A total of 483 target genes of alkaloids were identified through renowned databases. To establish a high-fidelity therapeutic network, we systematically isolated 342 overlapping differentially expressed genes (158 upregulated and 184 downregulated) that represent the direct functional intersection between *P. longum* alkaloid targets and ovarian cancer pathogenesis. Subsequent protein-protein interaction (PPI) analysis of these core targets revealed a highly interconnected and biologically robust network comprising 292 nodes and 6,373 edges. The immense statistical significance of this network (p = 1.0 × 10^−16^), combined with a high average local clustering coefficient (0.595), demonstrates that these targets execute a strongly coordinated systemic response rather than random, isolated protein associations. Through rigorous integration of six topological ranking algorithms via CytoHubba and MCODE cluster analysis (score >3), we successfully reduced the complexity of this signalling network down to ten key hub genes: TP53, CTNNB1, AKT1, IL6, TNF, EGFR, CASP3, BCL2, MYC, and JUN. The persistent topological rank of these nodes across various analytical criteria in this complex network confirms their influential roles in governing the survival and progression of ovarian cancer.

Gene ontology analysis elucidated that the molecular targets of *P. longum* alkaloids are mainly nuclear localized transcriptional and enzymatic regulators, densely packed with euchromatin and transcription complexes (Varadharajan et al., 2024). Through direct regulation of ubiquitin mediated protein degradation, kinase signalling, and DNA-binding, these hub genes rigidly key oncogenic processes, in particular apoptosis, cell population proliferation, and xenobiotic metabolism (Nan et al., 2019). Moreover, KEGG profiling identified their deep integration into diverse oncogenic signalling and drug resistance mechanisms, such as platinum and endocrine chemoresistance (Gong et al., 2014).

To confirm the pathological significance of the identified network, the differential expression of the ten core hub genes were assessed in human ovarian cancer samples through the GEPIA2 database. Following strict log2 (TPM+1) normalization, transcriptomic profiling highlighted the differential expression of five key genes (BCL2, EGFR, JUN, TNF, and TP53) in ovarian tumours as compared to their normal physiological controls (p ≤ 0.05). However, the other five genes (AKT1, CASP3, CTNNB1, IL6, and MYC) exhibited stable baseline expression. This unique differential expression pattern is highly significant; it not only confirms the direct role of BCL2, EGFR, JUN, TNF, and TP53 in oncogenic transformations of ovarian cancer and adverse clinical outcomes (Hossain et al., 2025; Wallis et al., 2023), but also in tumour immune evasion and inflammatory processes (Liu et al., 2025). moreover, the isolation of these genes as high priority targets for therapeutic intervention is highly relevant with recent pharmacological findings that demonstrates the ability of *P. longum* alkaloids, particularly piperlongumine, to reverse drug resistance driven by EGFR and to strictly regulate targeted protein degradation and apoptosis within the tumor microenvironment (Kung et al., 2021; Wang R et al., 2025).

To assess the clinical relevance of the differentially expressed hub genes, Kaplan-Meier survival analysis was performed to determine their prognostic value in predicting survival outcomes of ovarian cancer patients. JUN was the most significant adverse prognostic factor, with a hazard ratio (HR) of 1.40 (95% confidence interval [CI]: 1.23-1.58; p < 0.001), indicating that high expression levels are closely associated with poor survival outcomes. Similarly, EGFR showed a significant adverse effect (HR = 1.26, CI: 1.02-1.56; p = 0.03) on survival times. Importantly, these prognoses reflect recent independent transcriptomic profiling of ovarian cancer cohorts; for example, recent analyses of ovarian cancer survival using GEPIA (Gür et al., 2023) independently confirmed our findings, reporting identical adverse prognoses for both JUN (HR = 1.1) and EGFR (HR = 1.1) in addition to other major oncogenes, such as MYC. Conversely, while survival analysis initially indicated that higher bulk TP53 mRNA expression correlated with improved overall survival (HR = 0.76, CI: 0.76-0.87; p = 5.3e-05), this finding must be interpreted cautiously. Because high-grade serous ovarian cancer is defined by near-universal TP53 mutations, prognostic outcomes are fundamentally dictated by the functional consequence of the specific mutation type (e.g., gain-of-function versus loss-of-function) rather than baseline mRNA expression levels. Therefore, further functional validation distinguishing wild-type from mutant TP53 activity is required before clinical interpretations can be drawn, alongside TNF (HR = 0.81, CI: 0.71-0.92; p = 0.0016) and BCL2 (HR - = 0.87, CI: 0.76-0.99; p = 0.034). Likewise, the protective association of BCL2 transcript levels often reflect less malignant, high-grade ovarian cancer subtypes with improved therapeutic responses (Olbromski et al., 2023).

To explore the underlying genomic instability of the identified therapeutic targets, the mutational landscape of the hub genes in ovarian cancer was profiled using the GSCA database, which shows that TP53 has the highest mutation frequency of 100%, In contrast, EGFR, TNF, MYC, and JUN showed low mutation frequencies, while CASP3 and CTNNB1 had no mutations, suggesting their roles may be non-genetic. In addition, the fact that all samples analysed contained at least one genetic alteration highlights the potential of these hub genes as biomarkers and therapeutic targets in ovarian cancer, with TP53 as the main driver and others playing an important role through downstream pathways.

The type of genetic alteration of TP53 plays an important role in tumour development and influencing therapeutic treatment outcomes in ovarian cancer. In line with known genomic profiles, around 80% of somatic TP53 alterations are noted as missense mutations that are primarily located in the central, sequence-specific DNA binding domain, and often clustered around a few hotspot amino acid residues (Chen et al., 2022).

The epidermal growth factor receptor (EGFR) is a key transmembrane receptor which promotes tumour growth, epithelial-mesenchymal transition, and apoptosis inhibition through the hyperactivation of key intracellular signalling pathways, such as the PI3K/AKT1/mTOR and RAS/MAPK pathways. Dysregulated expression and somatic mutations of EGFR are well implicated in the development of a wide range of cancers, such as glioblastoma, breast, and ovarian cancers, where the receptors dynamically traffic not only to the plasma membrane but also to internal organelles (mitochondria and the nucleus) to drive tumour progression (Bortot et al., 2022).

TNF (Tumour Necrosis Factor) is a multifunctional cytokine, displaying a diverse range of tumour-promoting and tumour-supressing roles. In particular, TNF operates as a master manipulator of the tumour microenvironment (TME); by actively rewriting the composition of the local tissue environment, directing new vascular formation, and regulating the influx of immune cells, it effectively creates a niche that’s conductive to tumour growth (Alim et al., 2024). In the case of ovarian carcinoma, molecular profiling data shows that TNF-α levels are uniquely expressed, a pathogenic process which is highly dependent on a rigorous dependence on a positive feedback loop where TNF- α promotes its own expression (Korbecki et al., 2023).

MYC oncoprotein is a key driver of ovarian cancer gene expression. c-MYC acts a master transcription factor, it performs key orchestrations of fundamental oncogene processes, promoting upregulated cell proliferation, evasion of the apoptotic pathway, and metastasis, with its dysregulation strictly linked to increased tumour aggressiveness and poor patient prognosis (Tang et al., 2025).

C-JUN signalling pathway serves as a molecular checkpoint to the survival of tumour cells. Through targeted interactions with downstream protein substrates, this signalling pathway is a dynamic and tightly regulated component of fundamental oncogenic cellular events, in particular tumour cell proliferation and apoptosis (Shen et al., 2026).

While docking results showed paclitaxel (reference drug) displayed a significant and high binding affinity towards the target proteins (−12.47 kcal/mol was observed with EGFR), ADMET results predicted several violations of Lipinski’s rule of five for paclitaxel, along with poor gastrointestinal absorption, and blood-brain barrier permeability. Among the natural ligands, Piperine showed substantial binding affinity with EGFR (−8.70 kcal/mol), a promising target to improve ovarian cancer therapeutic efficacy. These findings reflect predicted binding interactions and therefore require additional experimental validation to confirm their functional significance. The docking scores obtained in this study were compared with previously reported molecular interactions of *P. longum* alkaloids. Piperine exhibits highly comparable binding energy (−8.70 kcal/mol) for piperine against glucose 1-dehydrogenase/hexose-6-phosphate dehydrogenase (H6PD) (Francis et al., 2024). Similarly, previous *in silico* evaluations have demonstrated that piperlonguminine binds to SRC with an affinity of −7.22 kcal/mol (Singh et al., 2023). Furthermore, the therapeutic relevance of piperlongumine is heavily supported by its reported strong interaction with the mammalian target of rapamycin (mTOR), exhibiting a binding energy of −8.7 kcal/mol (Du et al., 2025). Collectively, this evidence corroborates the biological plausibility of our modelled interactions, providing a strong theoretical foundation for future *in-vitro* and *in-vivo* validations. Along with the docking results, the MD simulation showed that the EGFR-piperine complex takes the most compact and stable structure, driven by strict hydrophobic anchoring (Sundaram et al., 2025). In contrast TP53 and TNF displayed intermediate stabilization (Bahena Culhuac and Bello, 2024), and JUN-piperlonguminine maintained dynamic, solvent-exposed conformations stabilized by sustained hydrogen bonding (Joshi et al., 2025).

## 5 Conclusion

This research demonstrates the value of a systematic and robust multi-faceted computational approach that integrates traditional phytochemistry and contemporary drug development to assess the ovarian cancer therapeutic potential of *P. longum* alkaloids (piperine, piperlongumine, and piperlonguminine). Through the integration of transcriptomic mining of five independent GEO datasets, network pharmacology, protein-protein interactome (PPI) topology, functional analysis, survival profiling and mutation analysis, we confirmed ten high-confidence hub genes (TP53, EGFR, JUN, TNF, BCL2, AKT1, CASP3, CTNNB1, IL6, and MYC as the key therapeutic targets of *P. longum* alkaloids in ovarian cancer. Based on differential expression, survival and independent prognostic hazard, BCL2, EGFR, JUN, TNF, and TP53 were clinically confirmed and further narrowed down. Molecular docking and molecular dynamic simulations unequivocally identified the most complementary interaction of piperine with EGFR (−8.70 kcal/mol) confirmed the highest stability, compactness and biological relevance of the EGFR-piperine complex within our tested complexes, by anchoring through hydrophobic interactions within the ATP binding cleft. Importantly, all the three alkaloids exhibit superior pharmacokinetic parameters and adhere to Lipinski rule of five compared to the conventional drug, paclitaxel, which reinforces their efficacy and oral-bioavailability as translational leads. Overall, our results extend a computationally validated and validated mechanistic lead for ovarian cancer therapy with *P. longum* alkaloids, providing a translatable multi-omics framework for natural alkaloid repurposing for therapy resistant cancers. *In vitro* and *in vivo* studies should be designed to experimentally validate these predictions, and for alkaloids to progress to preclinical testing.

## Data Availability

The datasets presented in this study can be found in online repositories. The names of the repository/repositories and accession number(s) can be found in the article/supplementary material.
